# Supplementary material for: Modeling, optimization, and comparable efficacy of T cell and hematopoietic stem cell gene editing for treating hyper‐IgM syndrome
Source: EMBO Mol Med. 2021 Jan 21;13(3):e13545. doi: 10.15252/emmm.202013545 (PMC7933961; doi:10.15252/emmm.202013545)
Supplement: Supplementary file 1 — Appendix [file EMMM-13-e13545-s001.pdf]

APPENDIX

Appendix Figure S1. Donor CD4+ cells engraftment in lymphoid organs after adoptive transfer of *ex vivo* cultured T cells.

Appendix Table S1. Off-target analysis of the selected *CD40LG* gRNA.

Appendix Table S2. Detection of *P. murina* infection by immunofluorescence, immunohistochemistry and ddPCR after HSPC therapy

Appendix Table S3. Detection of *P. murina* infection by immunofluorescence, immunohistochemistry and ddPCR after T-cell therapy.

Appendix Table S4. List of antibodies used in this study.

Appendix Table S5. List of primers used in this study.

Appendix Table S6. List of gRNAs tested in this study.

Supplementary Statistical Methods. Details of statistical analyses.

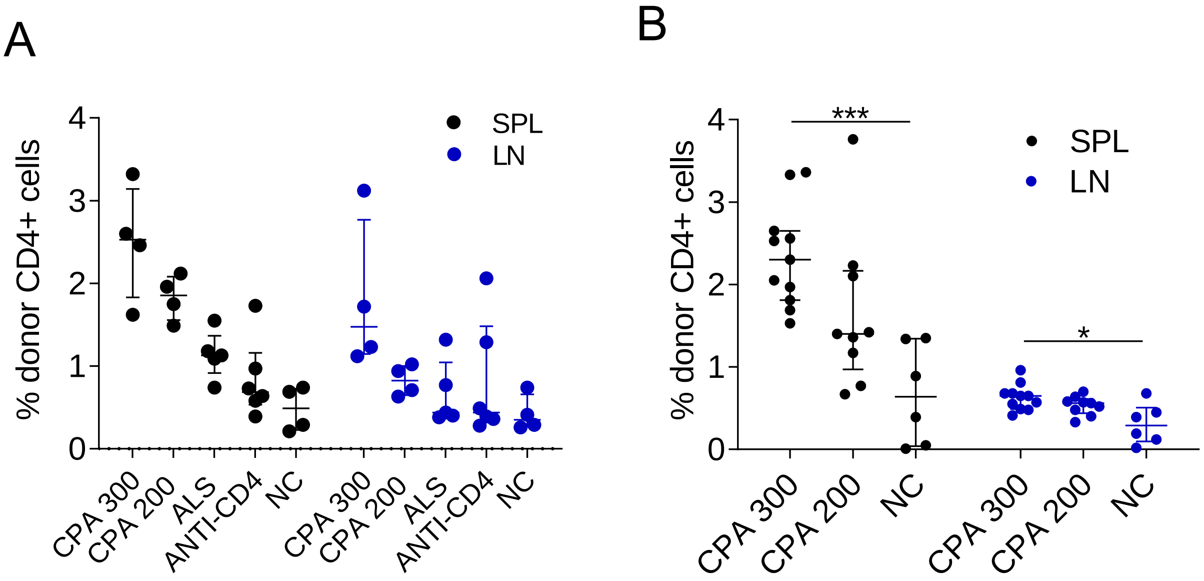

Appendix Figure S1. Donor CD4+ cells engraftment in lymphoid organs after adoptive transfer of *ex vivo* cultured T cells.

A Percentage of engrafted donor CD45.1+ cells in spleen and lymph nodes of mice from Fig 6B (n=4 NC, 4 CPA 300, 4 CPA 200, 5 ALS, 6 ANTI-CD4). Median ± IQR.

B Percentage of engrafted primed donor CD45.1+ cells in spleen and lymph nodes of mice from Fig 6F (n=6 NC, 11 CPA 300, 9 CPA 200). Kruskal-Wallis test followed by post-hoc analysis with Dunn's test. P-values were adjusted with Bonferroni's correction to account for multiple comparisons (*p=0.0213 and *** p=0.0007). Median ± IQR.

Appendix Table S1. Off-target analysis of the selected *CD40LG* gRNA.

**OT#***	**Location**	**Guide-Seq**	**Digenome**	**In silico**	**Amp-seq**	**Average off-target editing**
OT-1	Chr8: ~300 kb from gene	Yes	Yes	No	3/3 donors	0.24%
OT-2	Chr5: Intron of DMXL1	Yes	Yes	Yes	1/3 donors	0.13%
OT-3	Chr8: Intron of SPIDR	Yes	Yes	No	No	n.d.
OT-4	Chr6: >50 kb from gene	Yes	Yes	No	No	n.d.
OT-5	Chr15: ~30 kb from gene	No	Yes	No	No	n.d.
OT-6	Chr7: ~200 kb from gene	No	Yes	No	No	n.d.

*Top six of 93 analyzed putative off-targets of *S.p.* g1 gRNA. Full list in Dataset S1. N.d.: not detectable.

Appendix Table S2. Detection of *P. murina* infection by immunofluorescence, immunohistochemistry and ddPCR after HSPC therapy

**Group**	**IF for *P. murina* on lung homogenates**	**IHC for *P. murina* on histological sections of lungs**	**ddPCR**	**Histopathology (H&E): *P. murina*-associated interstitial pneumonia**
100% WT	-	-	**0.046**	-
	-	-	**0**	-
	-	-	**0**	-
	-	-	**0.009**	-
	-	-	**0**	-
	-	-	**0.039**	-
	+	-	**0.029**	-
	-	-	**0**	-
10% WT	-	-	**0.03**	-
	+++	+++	**14.987**	+
	+	+	**0.567**	-
	+	-	**0.013**	-
	-	-	**0.01**	-
	-	-	**0.005**	-
	-	-	**0**	-
25% WT	-	-	**0.089**	-
	+++	+++	**38.564**	+++
	-	-	**0.167**	-
	+	+	**0.238**	-
	+	-	**0.272**	-
	-	-	**0.007**	-
	+	+	**0.419**	+
0% WT	+	-	**1.943**	-
	+	+	**4.379**	+
	+++	+++	**91.03**	+++
	+	+	**0.064**	-
	-	-	**0.01**	-
	+++	+++	**121**	+++
	+	-	**0.512**	-
	+	+	**0.856**	+

Appendix Table S3. Detection of *P. murina* infection by immunofluorescence, immunohistochemistry and ddPCR after T-cell therapy.

Group	IF for *P. murina* on lung homogenates	IHC for *P. murina* on histological sections of lungs	ddPCR	Histopathology (H&E): *P. murina* associated interstitial pneumonia
+ T cells	-	-	**0.008**	-
	-	-	**0.013**	-
	-	-	**0.003**	-
	-	-	**0.001**	-
	-	-	**0.003**	-
	-	-	**0.002**	-
	-	-	**0.023**	-
	-	-	**0.002**	-
	-	-	**0.015**	-
CD40LG	+++	+++	**18.498**	+++
	-	-	**0.013**	-
	++	+++	**28**	+++
	-	-	**1.555**	-
	+++	++	**10.616**	-
	nd	+++	**180**	+++
	+++	++	**6.826**	+
	-	+	**0.036**	-

* Immunofluorescence on homogenate from lung tissue

**amount of positive material with morphological pattern compatible with P. murina

Scoring system: - = absent; + = minimal; ++ = moderate; +++ severe (abundant); nd: not determined because of artifacts

Appendix Table S4. List of antibodies used in this study.

Antibody	Fluorochrome	Clone	Company
**Anti-human antibodies**			
CD16/32	none		Miltenyi Biotec
CD271 (LNGFR)	APC	ME20.4-1.H4	Miltenyi Biotec
CD271 (LNGFR)	PE	ME20.4-1.H4	Miltenyi Biotec

CD271 (LNGFR)	PB	ME20.4-1.H4	Miltenyi Biotec
Anti-hEGFR	Biotin conjugated	#HU1	R&D Systems
Anti-biotin	APC	Bio3-18E7	Miltenyi Biotec
Anti-biotin	FITC	Bio3-18E7	Miltenyi Biotec
CD154	PE	24-31	Invitrogen
CD62L	PE	DREG-56	BD
CD62L	APC	DREG-56	Biolegend
CD45RA	PB	T6D11	Miltenyi Biotec
CD45RA	APC	HI100	Biolegend
CD45RA	FITC	L48	BD
CD133/2	PE	293C3	Miltenyi Biotec
CD34	PB	AC136	Miltenyi Biotec
CD34	PECy7	8G12	BD
CD90	APC	5E10	BD
CD45	PB	HI30	Biolegend
CD45	APCH7	HI30	eBioscience
CD19	PE	HIB19	BD
CD19	FITC	4G7	BD
CD19	PECy7	HIB19	Biolegend
CD19	APC	HIB19	Biolegend
CD19	PE-Vio770	SJ25C1	BD

CD3	PEcy7	HIT3a	Biolegend
CD3	APC	UCHT1	BD
CD3	FITC	BW264/56	Miltenyi Biotec
CD13	APC	WM15	BD
CD33	PECy7	P67.6	BD
CD38	Percp5.5	HB7	Biolegend
CD4	PB	RPA-T4	BD
CD4	PerCP	VIT4	Miltenyi Biotec
CD8	APCH7	SK1	BD
CD69	APC-Vio770	FN50	Miltenyi Biotec
CD25	APC	M-A251	Biolegend
CD197 (CCR7)	FITC	REA546	Miltenyi Biotec
CD45RO	PEC7	UCHL1	Biolegend
CD95	APC-Vio770	REA738	Miltenyi Biotec
IgM	PB		Biolegend
IgG	PE		Jackson ImmunoResearch
**Anti-mouse antibodies**			
CD16/32	none	2.4G2	BD
CD11b	APC-Cy7	M1/70	Biolegend
CD150	APC	TC15-12F12.2	Biolegend

CD19	PECy7	6D5	Biolegend
CD19	PB	6D5	Biolegend
CD25	Percp5.5	PC61	BD
CD25	APC	PC61	BD
CD3	PE	145-2C11	BD
CD8a	APC780	53-6.7	eBioscience
CD4	PB	RM4-5	BD
CD44	PECy7	IM7	BD
CD62L	APC	MEL-14	BD
CD45RA/B220	PE	RA3-6B2	BD
CD45RA/B220	PB	RA3-6B2	BD
Lineage Cocktail	PE		Biolegend
Sca1	PECY7	D7	BD
GL7	APC	1D3	eBioscience
PNA	FITC		Vector Laboratories
CD45.1	FITC	A20	BD
CD45.2	Percp5.5	104	BD

Appendix Table S5. List of primers used in this study.

**Description**	**Orientation**	**Sequence (5'-3')**
dsDNA donor template linearization	FW	GCAGGAACAAAAGAGCAGAG
	RV	ACCATTTTCTTGCTTTAAGAGTAG

NHEJ *CD40LG*	FW	CTTCACAAGCACTGATTGTAGTTGC
	RV	CCAAACACAAATAACCAACCAGACC
CD40LG integrations with 3' UTR as right homology arm	FW	TACATCTTTCGGACTGCTGAAG
	RV	ACTGAATAGGCTAGCACATCATC
CD40LG 5' HDR intregration junction ddPCR (except for different splice acceptors constructs)	FW	TTAGGAGGGGGTCTGATACA
	RV	TCCTCGATCTGTGGGAGGAAGAGAA
	Probe (FAM)	TCAGTCTCCCTCTGAGATGT
CD40LG 5' HDR integration junction ddPCR (different splice acceptors constructs)	FW	TCCGCTGCCAGATCTCTCGA
	RV	TCCTCGATCTGTGGGAGGAAGAGAA
	Probe (FAM)	TCAGTCTCCCTCTGAGATGT
CD40LG wild-type mRNA detection ddPCR	FW	GCACATGTCATAAGTGAGGC
	RV	CCCATTTTCCAGGGTTACCA
	Probe (FAM)	ACAACATCTGTGTTACAG
CD40LG codon optimized mRNA (edited) detection ddPCR	FW	CCAGATGATTGGGTCAGCA
	RV	TCTTCATGAACACGAAGTCCT
	Probe (FAM)	ACAAGATCGAGGACGAGA
P. Murina Ribosomal RNA Detection	FW	ATGAGGTGAAAAGTCGAAAGGG
	RV	TGAGGTCTCAGATGAAAAACCTCTT
	Probe (FAM)	AACAGCCCAGAATAATGAATAAA
OT chr8 – First step PCR	FW	GTTAATGGGTGTTTATTTCACTATTCTTGC
	RV	TGAAACTATCTCTGTGTTGGTTATAAAGC

OT chr8 - P5 NGS 1	FW	AATGATACGGCGACCACCGAGATCTACACTAGATCGCNNWNNWNNACACTCTTTCCCTACACGACGCTCTTCCGATCT GTTAATGGGTGTTTATTTCACTATTCTTGC
OT chr8 - P5 NGS 2	FW	AATGATACGGCGACCACCGAGATCTACACCTCTCTATNNWNNWNNACACTCTTTCCCTACACGACGCTCTTCCGATCT GTTAATGGGTGTTTATTTCACTATTCTTGC
OT chr8 - P5 NGS 3	FW	AATGATACGGCGACCACCGAGATCTACACTATCCTCTNNWNNWNNACACTCTTTCCCTACACGACGCTCTTCCGATCT GTTAATGGGTGTTTATTTCACTATTCTTGC
OT chr8 - P5 NGS 4	FW	AATGATACGGCGACCACCGAGATCTACACAGAGTAGANNWNNWNNACACTCTTTCCCTACACGACGCTCTTCCGATCTGTTAATGGGTGTTTATTTCACTATTCTTGC
OT chr8 – P7 NGS 1	RV	CAAGCAGAAGACGGCATACGAGATTCGCCTTAGTGACTGGAGTCCTCTCTATGGGCAGTCGGTGATGAAACTATCTCTGTGTTGGTTATAAAGC
OT chr8 – P7 NGS 2	RV	CAAGCAGAAGACGGCATACGAGATCTAGTACGGTGACTGGAGTCCTCTCTATGGGCAGTCGGTGATGAAACTATCTCTGTGTTGGTTATAAAGC
OT chr8 – P7 NGS 3	RV	CAAGCAGAAGACGGCATACGAGATTTCTGCCTGTGACTGGAGTCCTCTCTATGGGCAGTCGGTGATGAAACTATCTCTGTGTTGGTTATAAAGC

OT chr8 – P7 NGS 4	RV	CAAGCAGAAGACGGCATACGAGATGCTCAGGA GTGACTGGAGTCCTCTCTATGGGCAGTCGGTGA TGAAACTATCTCTGTGTTGGTTATAAAGC
OT chr8 – P7 NGS 5	RV	CAAGCAGAAGACGGCATACGAGATAGGAGTCC GTGACTGGAGTCCTCTCTATGGGCAGTCGGTGA TGAAACTATCTCTGTGTTGGTTATAAAGC
OT chr8 – P7 NGS 6	RV	CAAGCAGAAGACGGCATACGAGATCATGCCTA GTGACTGGAGTCCTCTCTATGGGCAGTCGGTGA TGAAACTATCTCTGTGTTGGTTATAAAGC

Appendix Table S6. List of gRNAs tested in this study.

Name	Sequence	Cas9
g1	TGGATGATTGCACTTTATCA	Pyogenes
g2	TTTTCTAACAGGATAAGGTG	Pyogenes
g3	CAGTGGACTGATATTTACCG	Pyogenes
g4	AGTGAGGGCTGAAGTCATCCA	Aureus
g5	TGGGTTATCCAAATATTAGGT	Aureus
g6	CAATGAGAAATGTGACAATTA	Aureus
g7	AGAATAGCTCTGATTTCTACC	Aureus

Supplementary Statistical Methods. Details of statistical analyses.

Nonlinear mixed-effects model analysis of longitudinal data in Fig 5B and 5C

Due to the type of longitudinal trajectories of data in Fig 5B and 5C, the comparison of the longitudinal trend of the two groups was performed with a nonlinear mixed-effects (NLME) regression with the following asymptotic model:

$$\sqrt{Y} = Asym + (R0 - Asym)e^{(-Time*e^{lrc})},$$

where the time was considered as continuous with the origin shifted at day 1 (for a better interpretation of the parameters of the model), while the dependent variable Y was transformed in the square root scale to meet the assumption of normality of the residuals. In the model, the parameter $Asym$ represents the horizontal asymptote (i.e. the value of the plateau reached by $\sqrt{Y}$). The parameter $R0$ represents the value at day 1 and the lrc parameter is the natural logarithm of

the rate constant. For evaluating differences between the two groups, in the full model, all parameters were allowed to depend on the group variable:

$$Asym = \beta_0 + \beta_1(NC), R0 = \gamma_0 + \gamma_1(NC), lrc = \delta_0 + \delta_1(NC)$$

where $NC = 1$, for NC group, and $NC = 0$ for CPA 300 group. For each dependent variable, the final model was obtained with a backward variable selection of fixed-effects covariates (where p-values less than 0.05 were considered significant). Random effects were set on the asymptote to account for mice heterogeneity. When necessary few observations were excluded from the analysis since they were outliers for the full model.

<u>Final NLME model for n. recipient CD3+ cells data in Fig 5B (excluded 11 outlier observations from the analysis):</u>

$$Asym = \beta_0 + \beta_1(NC), R0 = \gamma_0 + \gamma_1(NC), lrc = \delta_0$$

Parameter	Estimate	p-value
β_0	1.6549	<0.0001
β_1	-0.3933	0.0297
γ_0	0.2328	0.0001
γ_1	1.0624	<0.0001
δ_0	-4.3562	<0.0001

<u>Final NLME model for n. donor T cells data in Fig 5C (excluded 8 outlier observations from the analysis):</u>

$$Asym = \beta_0 + \beta_1(NC), R0 = \gamma_0 + \gamma_1(NC), lrc = \delta_0 + \delta_1(NC)$$

Parameter	Estimate	p-value
β_0	0.4649	<0.0001
β_1	-0.2992	<0.0001
γ_0	0.1608	<0.0001
γ_1	0.1079	0.0001
δ_0	-2.2529	<0.0001
δ_1	-2.3329	0.0130

Linear mixed-effects model analysis of data in Fig 5F, 6E, 6H and 7C

For evaluating differences of IgG concentration among groups overall and, within each group, between pre and post values, a full linear mixed-effects (LME) model was estimated, followed by a post-hoc analysis with the R package phia. In each post-hoc analysis, p-values were adjusted with Bonferroni's correction to account for multiple comparisons or testing, depending on the analysis. The full LME model included the following terms: time (pre vs post), group and an interaction term between time and group. When necessary, to meet the assumptions of the LME model, an adequate transformation of the dependent variable was used (the natural logarithmic transformation for data of first boost in Fig 5F and 6E and data of Fig 7C and the square root transformation for data of second boost in Fig 6H). Random effects of the LME model were set on the intercept term and they were defined either to account only for the variability among mice in case of a single experiment (data of Fig 6H), or as nested to account for the variability among

experiments and among mice within each experiment (data of Fig 5F, 6E and 7C). Only the results of post-hoc analysis are reported.

Post-hoc analysis of IgG concentration of first boost in Fig 5F:

Post-hoc analysis testing the overall difference among groups:

Group comparison	Estimated difference	P-value
NC vs CPS 300	-2.0480	<0.0001
NC vs CD40LG	1.4230	<0.0001
NC vs WT	-4.4195	<0.0001
CPA 300 vs CD40LG	3.4711	<0.0001
CPA 300 vs WT	-2.3714	<0.0001
CD40LG vs WT	-5.8425	<0.0001

Post-hoc analysis testing the differences between pre vs post values for each group:

Group	Estimated difference	P-value
NC	0.8318	<0.0001
CPA 300	-0.1799	1.0000
CD40LG	0.6498	0.0201
WT	-0.7004	0.0100

Post-hoc analysis of IgG concentration of first boost in Fig 6E (excluded group CPA 200 from the analysis because n=4):

Post-hoc analysis testing the overall difference among groups:

Group comparison	Estimated difference	P-value
NC vs ALS	-0.7029	0.3639
NC vs ANTI-CD4	-1.2905	0.0002
NC vs CPA 300	-1.2066	<0.0001
NC vs CD40LG	1.9892	<0.0001
NC vs WT	-4.0915	<0.0001
ALS vs ANTI-CD4	-0.5876	1.0000
ALS vs CPA 300	-0.5037	1.0000
ALS vs CD40LG	2.6921	<0.0001
ALS vs WT	-3.3886	<0.0001
ANTI-CD4 vs CPA 300	0.0840	1.0000
ANTI-CD4 vs CD40LG	3.2797	<0.0001
ANTI-CD4 vs WT	-2.8010	<0.0001
CPA 300 vs CD40LG	3.1957	<0.0001
CPA 300 vs WT	-2.8850	<0.0001
CD40LG vs WT	-6.0807	<0.0001

Post-hoc analysis testing the differences between pre vs post values for each group:

Group	Estimated difference	P-value
UT	0.8956	<0.0001
ALS	1.0212	<0.0001
ANTI-CD4	0.6644	0.0022
CPA 300	-0.5643	<0.0001
CD40LG	1.0779	<0.0001
WT	-1.0489	<0.0001

Post-hoc analysis of IgG concentration of first boost in Fig 6H:

Post-hoc analysis testing the overall difference among groups:

Group comparison	Estimated difference	P-value
NC vs CD40LG	494.41	0.2545
NC vs CPA 300	-167.56	1.0000
NC vs CPA 200	-307.53	0.0787
CD40LG vs CPA 300	-661.96	0.0282
CD40LG vs CPA 200	-801.94	0.0037
CPA 300 vs CPA 200	-139.98	1.0000

Post-hoc analysis testing the differences between pre vs post values for each group:

Group	Estimated difference	P-value
NC	-141.570	0.5027
CD40LG	10.608	1.0000
CPA 300	-126.274	0.2576
CPA 200	-133.687	0.2009

Post-hoc analysis of IgG concentration of second boost in Fig 6H:

Post-hoc analysis testing the overall difference among groups:

Group comparison	Estimated difference	P-value
NC vs CPA 300	-3.0329	0.7276
NC vs CPA 200	-3.4306	0.6091
CPA 300 vs CPA 200	-0.3977	1.0000

Post-hoc analysis testing the differences between pre vs post values for each group:

Group	Estimated difference	P-value
NC	-10.028	0.0018
CPA 300	-11.853	<0.0001
CPA 200	-11.768	<0.0001

Post-hoc analysis of IgG concentration of first boost in Fig 7C:

Post-hoc analysis testing the overall difference among groups:

Group comparison	Estimated difference	P-value
0% vs 1%	-0.3086	1.0000
0% vs 10%	-2.8160	<0.0001
0% vs 25%	-3.3936	<0.0001
0% vs 100%	-4.4820	<0.0001
1% vs 10%	-2.5074	<0.0001
1% vs 25%	-3.0850	<0.0001
1% vs 100%	-4.1733	<0.0001
10% vs 25%	-0.5776	0.0511
10% vs 100%	-1.6660	<0.0001
25% vs 100%	-1.0884	<0.0001

Post-hoc analysis testing the differences between pre vs post values for each group:

Group	Estimated difference	P-value
0%	0.7441	<0.0001
1%	-0.3287	0.9528
10%	-0.0793	1.0000
25%	-0.6546	0.0001
100%	-1.0919	<0.0001

Linear mixed-effects model analysis of data in Fig 6B and 6C

For evaluating differences among groups at a fixed time-point accounting for data belonging to different experiments, a linear mixed-effects (LME) model with the only group term was estimated, followed by a post-hoc analysis comparing all pairs of groups with the R package phia. In each post-hoc analysis, p-values were adjusted with Bonferroni's correction to account for multiple comparisons. To meet the assumptions of the model, an adequate transformation of the dependent variable was used in the corresponding LME model (for data in Fig 6B the natural logarithmic scale, while for data in Fig 6C the square root transformation) and few observations were excluded from the analysis since they were outliers for model. Random effects of the LME model were set on the intercept term to account for the variability between experiments. Only the results of post-hoc analysis are reported.

Post-hoc analysis of n. recipient CD3+ cells at day 3\4 of Fig 6B (excluded 1 outlier observation from the analysis):

Group comparison	Estimated difference	P-value
NC vs CPA 300	3.3077	<0.0001
NC vs CPA 200	2.1906	<0.0001
NC vs ANTI-CD4	1.1168	<0.0001
NC vs ALS	1.8642	<0.0001
CPA 300 vs CPA 200	-1.1171	<0.0001
CPA 300 vs ANTI-CD4	-2.1910	<0.0001

CPA 300 vs ALS	-1.4436	<0.0001
CPA 200 vs ANTI-CD4	-1.0739	0.0009
CPA 200 vs ALS	-0.3265	1.0000
ANTI-CD4 vs ALS	0.7474	0.0630

<u>Post-hoc analysis of n. donor CD4+ cells at day 18\24 of Fig 6C (excluded group CPA 200, because n=4, and 1 outlier observation from the analysis):</u>

Group comparison	Estimated difference	P-value
NC vs CPA 300	-0.4122	<0.0001
NC vs ANTI-CD4	-0.1379	<0.0001
NC vs ALS	-0.1376	0.0002
CPA 300 vs ANTI-CD4	0.2743	<0.0001
CPA 300 vs ALS	0.2746	<0.0001
ANTI-CD4 vs ALS	0.0003	1.0000

Nonlinear mixed-effects model analysis of data in Fig 6D

The nonlinear relationship between n. recipient CD3+ cells at day 3\4 and n. donor CD4+ cells at day 18\24 was analyzed with a nonlinear mixed-effects (NLME) model analysis by using an asymptotic model. The standard NLME model with horizontal right asymptote, was reparametrized in the following way to enhance the interpretation of the results with respect to the aim of the analysis:

$$\sqrt{Y} = R0 - delta + delta * e^{(-X*e^{lrc})}.$$

where the X was n. recipient CD3+ cells at day 3\4, while the dependent variable Y (n. donor CD4+ cells at day 18\24) was transformed in the square root scale to meet the assumption of normality of the residuals. In the model, the parameter $R0$ represents the value at $X=0$ and the lrc parameter is the natural logarithm of the rate constant. The parameter *delta* represents the difference between *R0* and the value of the horizontal asymptote (i.e. the value of the plateau reached by $\sqrt{Y}$ for higher values of X). Thus, a positive value of the *delta* parameter denotes that a decrease of X corresponds to an increase of Y. Random effects were set on the *delta* parameter to account for heterogeneity among groups and among experiments. One observation was excluded from the analysis since it was an outlier for the full model.

Parameter	Estimate	P-value
R0	0.7132	<0.0001
delta	0.3274	0.0006
Lrc	2.4347	0.0003

Linear mixed-effects model analysis for longitudinal data in Fig 6F, 6G, 1H, 2F, EV4A, EV1J

When the nonlinear trajectories over time could not be modeled with known nonlinear mixed-effects models or data were not adequate for applying this kind of models, for evaluating

differences among groups over time, a full linear mixed-effects (LME) model was estimated, which included the following terms: time (treated as categorical variable), group and an interaction term between time and group. In case of two groups, results of the comparisons at each time-points were retrieved directly from the estimated LME model. In case of more than three groups, a post-hoc analysis with the R package phia was performed for testing differences between all pairs of groups at each time-point. In case of data in Fig 2F, also a post-hoc analysis for testing the overall differences among groups was performed. In each post-hoc analysis, p-values were adjusted with Bonferroni's correction to account for both multiple testing and comparisons. In this case, only the results of post-hoc analysis are reported. When necessary, to meet the assumptions of the model, an adequate transformation of the dependent variable was used in the corresponding LME model (the square root transformation for data in Fig 6F, the cubic root transformation for data in Fig 2F, the natural logarithmic transformation for RFI data in Fig 1H, RFI data in Fig EV1J, the log(x+0.01) transformation for data in Fig 6G due to the presence of zeros) and, eventually, few observations were excluded from the analysis since they were outliers for model. Random effects of the LME model were set on the intercept term and they were defined either to account only for the variability among mice (data of Fig 6F, 6G, 2F and EV4A), or as nested to account for the variability among donors and among samples with the same donor (data of Fig 1H and EV1J).

LME model of % donor T cells comparing CD40LG vs WT $2x10^6$ doses in Fig EV4A (time-points 19, 26, 33 days were excluded from the analysis since n=3 for WT):

Parameter	Estimate	P-value
Intercept	0.0640	<0.0001
at day 4 vs 1	-0.0100	0.1751
at day 7 vs 1	-0.0200	0.0119
group CD40LG vs WT	-0.0080	0.5209
at day 4:group CD40LG vs WT	0.0000	1.0000
at day 7:group CD40LG vs WT	0.0080	0.4339

LME model of % donor T cells comparing CD40LG vs WT $20x10^6$ doses in Fig EV4A:

Parameter	Estimate	P-value
Intercept	0.5700	<0.0001
at day 4 vs 1	-0.1040	0.2310
at day 7 vs 1	-0.0060	0.9444
at day 19 vs 1	-0.0960	0.2682
at day 26 vs 1	-0.2420	0.0072
at day 33 vs 1	-0.2000	0.0244
group CD40LG vs WT	0.0760	0.4358
at day 4:group CD40LG vs WT	-0.0460	0.7056
at day 7:group CD40LG vs WT	-0.1860	0.1319
at day 19:group CD40LG vs WT	-0.1760	0.1533
at day 26:group CD40LG vs WT	-0.0900	0.4610
at day 33:group CD40LG vs WT	-0.1460	0.2344

Post-hoc analysis testing the differences between all pairs of groups at each time-point of n. recipient CD3+ cells data in Fig 6F:

Day	Group comparison	Estimated difference	P-value
4	NC vs CPA 300	1.2606	<0.0001
4	NC vs CPA 200	0.8920	<0.0001
4	CPA 300 vs CPA 200	-0.3687	<0.0001
10	NC vs CPA 300	0.8721	<0.0001
10	NC vs CPA 200	0.7295	<0.0001
10	CPA 300 vs CPA 200	-0.1426	0.8994
25	NC vs CPA 300	-0.4551	<0.0001
25	NC vs CPA 200	-0.3542	0.0003
25	CPA 300 vs CPA 200	0.1009	1.0000
52	NC vs CPA 300	0.1225	1.0000
52	NC vs CPA 200	0.0280	1.0000
52	CPA 300 vs CPA 200	-0.0945	1.0000
128	NC vs CPA 300	-0.1935	0.3338
128	NC vs CPA 200	0.0194	1.0000
128	CPA 300 vs CPA 200	0.2129	0.0618
215	NC vs CPA 300	-0.4329	<0.0001
215	NC vs CPA 200	0.1655	1.0000
215	CPA 300 vs CPA 200	0.5984	<0.0001

Post-hoc analysis testing the differences between all pairs of groups at each time-point of n. donor CD4+ cells data in Fig 6G:

Day	Group comparison	Estimated difference	P-value
4	NC vs CPA 300	0.6067	0.0882
4	NC vs CPA 200	0.1950	1.0000
4	CPA 300 vs CPA 200	-0.4117	0.4151
10	NC vs CPA 300	-1.8595	<0.0001
10	NC vs CPA 200	-1.2032	<0.0001
10	CPA 300 vs CPA 200	0.6563	0.0086
25	NC vs CPA 300	-1.9468	<0.0001
25	NC vs CPA 200	-1.6638	<0.0001
25	CPA 300 vs CPA 200	0.2830	1.0000
52	NC vs CPA 300	-1.8021	<0.0001
52	NC vs CPA 200	-1.2322	<0.0001
52	CPA 300 vs CPA 200	0.5699	0.0498
128	NC vs CPA 300	-1.5464	<0.0001
128	NC vs CPA 200	-0.8890	0.0010
128	CPA 300 vs CPA 200	0.6574	0.0124

215	NC vs CPA 300	-1.6429	<0.0001
215	NC vs CPA 200	-0.6324	0.0751
215	CPA 300 vs CPA 200	1.0105	<0.0001

LME model of RFI in Fig 1H:

**Parameter**	**Estimate**	**P-value**
Intercept	6.4269	<0.0001
at hour 3 vs 0	1.2568	<0.0001
at hour 8 vs 0	1.3998	<0.0001
at hour 24 vs 0	0.2025	0.0016
at hour 48 vs 0	0.1870	0.0034
Group HD GFP+ vs GFP-	0.0000	1.0000
at hour 3:Group HD GFP+ vs GFP-	-0.3459	0.0002
at hour 8:Group HD GFP+ vs GFP-	-0.4433	<0.0001
at hour 24:Group HD GFP+ vs GFP-	-0.1609	0.0692
at hour 48:Group HD GFP+ vs GFP-	-0.2029	0.0230

LME model of %CD40LG+ cells in Fig 1H (excluded 3 outlier observations from the analysis):

**Parameter**	**Estimate**	**P-value**
Intercept	3.7723	0.0135
at hour 3 vs 0	69.2857	<0.0001
at hour 8 vs 0	76.5891	<0.0001
at hour 24 vs 0	16.9704	<0.0001
at hour 48 vs 0	17.4771	<0.0001
Group HD GFP+ vs GFP-	-2.9604	0.1033
at hour 3:Group HD GFP+ vs GFP-	-6.0205	0.0090
at hour 8:Group HD GFP+ vs GFP-	-4.4848	0.0450
at hour 24:Group HD GFP+ vs GFP-	-7.7445	0.0008
at hour 48:Group HD GFP+ vs GFP-	-9.3037	0.0001

Post-hoc analysis testing the differences between all pairs of groups at each time-point of CD4+ RFI data of case (i) in Fig EV1J:

**Hour**	**Group comparison**	**Estimated difference**	**P-value**
0	CO GFP+ vs WT GFP+	0.1461	1.0000
0	CO GFP+ vs GFP-	0.0763	1.0000
0	WT GFP+ vs GFP-	-0.0698	1.0000
3	CO GFP+ vs WT GFP+	0.2019	0.2179
3	CO GFP+ vs GFP-	-0.3724	<0.0001

3	WT GFP+ vs GFP-	-0.5742	<0.0001
6	CO GFP+ vs WT GFP+	0.3086	0.0022
6	CO GFP+ vs GFP-	-0.5215	<0.0001
6	WT GFP+ vs GFP-	-0.8301	<0.0001
8	CO GFP+ vs WT GFP+	0.2927	0.0049
8	CO GFP+ vs GFP-	-0.4872	<0.0001
8	WT GFP+ vs GFP-	-0.7799	<0.0001
24	CO GFP+ vs WT GFP+	0.0561	1.0000
24	CO GFP+ vs GFP-	-0.1042	1.0000
24	WT GFP+ vs GFP-	-0.1602	0.3071
48	CO GFP+ vs WT GFP+	-0.0175	1.0000
48	CO GFP+ vs GFP-	-0.0605	1.0000
48	WT GFP+ vs GFP-	-0.0430	1.0000

Post-hoc analysis testing the differences between all pairs of groups at each time-point of RFI data of case (iv) in Fig EV1J:

Hour	Group comparison	Estimated difference	P-value
0	CD40LG GFP+ vs GFP-	-0.0037	1.0000
0	CD40LG GFP+ vs SV40 GFP+	-0.0378	1.0000
0	GFP- vs SV40 GFP+	-0.0341	1.0000
3	CD40LG GFP+ vs GFP-	-0.4446	<0.0001
3	CD40LG GFP+ vs SV40 GFP+	0.0334	1.0000
3	GFP- vs SV40 GFP+	0.4780	<0.0001
6	CD40LG GFP+ vs GFP-	-0.6614	<0.0001
6	CD40LG GFP+ vs SV40 GFP+	0.0049	1.0000
6	GFP- vs SV40 GFP+	0.6662	<0.0001
8	CD40LG GFP+ vs GFP-	-0.6120	<0.0001
8	CD40LG GFP+ vs SV40 GFP+	0.0193	1.0000
8	GFP- vs SV40 GFP+	0.6312	<0.0001
24	CD40LG GFP+ vs GFP-	-0.0990	1.0000
24	CD40LG GFP+ vs SV40 GFP+	0.0424	1.0000
24	GFP- vs SV40 GFP+	0.1414	1.0000
48	CD40LG GFP+ vs GFP-	-0.0280	1.0000
48	CD40LG GFP+ vs SV40 GFP+	0.0236	1.0000
48	GFP- vs SV40 GFP+	0.0516	1.0000

Post-hoc analysis testing the differences between all pairs of groups at each time-point of %hCD45+ cells data in Fig 2F:

Week	Group comparison	Estimated difference	P-value

1	UT vs NGFR+	-0.3447	1.0000
1	UT vs NGFR bulk	0.2016	1.0000
1	NGFR+ vs NGFR bulk	0.5463	1.0000
4	UT vs NGFR+	-0.0597	1.0000
4	UT vs NGFR bulk	0.7091	1.0000
4	NGFR+ vs NGFR bulk	0.7687	0.9287
6	UT vs NGFR+	-0.4633	1.0000
6	UT vs NGFR bulk	0.0874	1.0000
6	NGFR+ vs NGFR bulk	0.5506	1.0000
8	UT vs NGFR+	-0.2373	1.0000
8	UT vs NGFR bulk	-0.2213	1.0000
8	NGFR+ vs NGFR bulk	0.0160	1.0000
10	UT vs NGFR+	-0.3650	1.0000
10	UT vs NGFR bulk	-0.3690	1.0000
10	NGFR+ vs NGFR bulk	-0.0040	1.0000
12	UT vs NGFR+	-0.1739	1.0000
12	UT vs NGFR bulk	-0.7592	0.9855
12	NGFR+ vs NGFR bulk	-0.5853	1.0000
14	UT vs NGFR+	0.0877	1.0000
14	UT vs NGFR bulk	-0.6510	1.0000
14	NGFR+ vs NGFR bulk	-0.7388	1.0000

Post-hoc analysis testing the overall differences between all pairs of groups of %hCD45+ cells data in Fig 2F:

Group comparison	Estimated difference	P-value
UT vs NGFR+	-0.2223	1.0000
UT vs NGFR bulk	-0.1432	1.0000
NGFR+ vs NGFR bulk	0.0791	1.0000

Linear mixed-effects model analysis of longitudinal data in Fig 2G and 4E

Due to the linear longitudinal trajectories of data in Fig 2G and 4E, the comparison of the longitudinal trend among groups was performed with a linear mixed-effects (LME) with time treated as a continuous variable. In order to test eventual differences among the groups, firstly a full model was estimated including the following terms: time, group and an interaction term between time and group (in case of the data of Fig 4E, the origin of the time was shifted at week 6 for a better interpretation of the parameters of the model). Then, the final model was obtained with a backward variable selection of fixed-effects covariates (where p-values less than 0.05 were considered significant). Random effects were set on the intercept to account for mice heterogeneity. In case of the analysis of the data in Fig 4E, also a post-hoc analysis was performed

with the R package phia for testing the differences between all pairs of groups both at 20 weeks and with respect to the slope of the model.

Final LME model of % editing by ddPCR (called *Y*) in Fig 2G:

$$Y = \beta_0 + \beta_1(Bulk),$$

where $Bulk = 1$, for NGFR bulk group, and $Bulk = 0$ for NGFR+ group.

Parameter	Estimate	p-value
β_0	75.3772	<0.0001
β_1	-45.1728	<0.0001

Final LME model of % HDR (called *Y*) in Fig 4E:

$$Y = \beta_0 + \beta_1(NGFR) + \beta_2(NGFR + GSE56) + \gamma_0 Time + \gamma_1 Time * (NGFR) + \gamma_2 Time * (NGFR + GSE56),$$

where $NGFR = 1$, for NGFR group, and $NGFR = 0$ otherwise, $NGFR + GSE56 = 1$, for NGFR+GSE56 group, and $NGFR + GSE56 = 0$ otherwise.

Parameter	Estimate	P-value
β_0	33.5432	<0.0001
β_1	-10.5454	0.0019
β_2	-12.3534	0.0008
γ_0	-0.3305	0.0588
γ_1	-0.7397	0.0028
γ_2	0.4782	0.0590

Post-hoc analysis testing the differences between all pairs of groups at time-point 20 weeks of % HDR data in Fig 4E:

Group comparison	Estimated difference	P-value
NR+GSE56 vs NGFR+GSE56	5.6582	0.0812
NR+GSE56 vs NGFR	20.9006	<0.0001
NGFR+GSE56 vs NGFR	15.2424	<0.0001

Post-hoc analysis testing the differences between all pairs of groups in the slope of the LME model of % HDR data in Fig 4E:

Group comparison	Estimated difference	P-value
NR+GSE56 vs NGFR+GSE56	-0.4782	0.1624
NR+GSE56 vs NGFR	0.7397	0.0054
NGFR+GSE56 vs NGFR	1.2179	<0.0001

Nonlinear mixed-effects model analysis of longitudinal data in Fig 4D

Due to the type of longitudinal trajectories of data in Fig 4D, the comparison of the longitudinal trend among the groups was performed with a nonlinear mixed-effects (NLME) model regression with the following asymptotic model:

$$\%\text{hCD45+ cells} = Asym + (R0 - Asym)e^{(-Time * e^{lrc})},$$

where the time was considered as continuous with the origin shifted at week 6 (for a better interpretation of the parameters of the model). In the model, the parameter $Asym$ represents the horizontal asymptote (i.e. the value of the plateau reached by the dependent variable). The parameter $R0$ represents the value at week 6 and the lrc parameter is the natural logarithm of the rate constant. For evaluating differences among groups, in the full model, all parameters were allowed to depend on the group variables:

$$Asym = \beta_0 + \beta_1(NGFR + GSE56) + \beta_2(NR + GSE56),$$

$$R0 = \gamma_0 + \gamma_1(NGFR + GSE56) + \gamma_2(NR + GSE56), lrc = \delta_0 + \delta_1(NGFR + GSE56) + \delta_2(NR + GSE56)$$

where $NGFR + GSE56 = 1$, for NGFR+GSE56 group, and $NGFR + GSE56 = 0$ otherwise, $NR + GSE56 = 1$, for NR+GSE56 group, and $NR + GSE56 = 0$ otherwise. The final model was obtained with a backward variable selection of fixed-effects covariates (where p-values less than 0.05 were considered significant). Random effects were set on the asymptote to account for mice heterogeneity.

<u>Final NLME model:</u>

$$Asym = \beta_0,$$

$$R0 = \gamma_0 + \gamma_1(NGFR + GSE56) + \gamma_2(NR + GSE56), lrc = \delta_0 + \delta_1(NGFR + GSE56) + \delta_2(NR + GSE56)$$

**Parameter**	**Estimate**	**P-value**
$Asym$	74.5959	<0.0001
γ_0	3.8121	0.3570
γ_1	14.9799	0.0213
γ_2	17.7147	0.0069
δ_0	-1.7940	<0.0001
δ_1	1.0127	0.0034
δ_2	0.9707	0.0080
