# Supplementary material for: Modeling, optimization, and comparable efficacy of T cell and hematopoietic stem cell gene editing for treating hyper‐IgM syndrome
Source: EMBO Mol Med. 2021 Jan 21;13(3):e13545. doi: 10.15252/emmm.202013545 (PMC7933961; doi:10.15252/emmm.202013545)
Supplement: Supplementary file 2 — Expanded View Figures PDF [file EMMM-13-e13545-s002.pdf]

Expanded View Figures

Figure EV1. Optimization of the *CD40LG* gene correction strategy.

A Schematics of *Streptococcus pyogenes* (*S.p.;* g1–g3) and *Staphylococcus aureus* (*S.a.;* g4–g7) Cas9 gRNAs targeting *CD40LG* intron 1.
B Percentage of insertion and deletions after *CD40LG* editing with gRNAs from (A), measured by Ill-Seq (n = 2 g3, 8 g7, 6 for other groups). Median ± IQR.
C Percentage of ssODN-mediated targeted integration after *CD40LG* editing with gRNAs from (A), measured by Ill-Seq (n = 4, except g3, n = 2). Median ± IQR.
D Percentage of AAV6-mediated HDR after *CD40LG* editing with g1 or g4 from (A), measured by ddPCR (n = 10 g1, 4 g4). Median ± IQR.
E Venn Diagram representing g1 candidate off-target sites identified by in silico prediction, Digenome-Seq and Guide-Seq.
F Percentage of deletion at the off-target cut site in UT cells (n = 3) and cells edited with WT Cas9 or HF Cas9. T cells from three different male donors were edited with two doses (1.25 and 2.5 μM) of WT Cas9 or HF Cas9 (n = 3 for each dose), while cord blood-derived $CD34^+$ cells from three different male donors were edited with 2.5 μM WT Cas9 or HF Cas9 (n = 3). The comparison of the frequency of T cells between WT and HF was performed with an LME model accounting for the different doses included in the analysis and with random effects defined to account for the same donor (n = 6) (**P = 0.0027). Median.
G Percentage of on-target integration at 17 days after *CD40LG* editing in T cells from (F), measured by FACS, and at 4 days after *CD40LG* editing in HSPC from (F), measured by molecular analysis (ddPCR) (n = 3). Median ± Range.
H Upper panel: Schematics of donor DNA templates with different splice acceptor (SA) sequences: (i) HBB SA, derived from introns of *HBB* gene, (ii) EF1a SA, derived from the first intron of *EEF1A1* gene, and (iii) Synthetic (Synth) SA derived from consensus sequence of SA. Lower panel: Representative plots showing CD40L and GFP expression after PMA/Ionomycin stimulation in $CD4^+$ T cells, edited with the three constructs described above and delivered to the cells as linear double-strand DNA or as integrase defective lentiviral vectors.
I Bar plot showing the percentage of GFP^+ cells or HDR after *CD40LG* editing in T cells from (H) (n = 1).
J Time course of CD40L surface expression after PMA/Ionomycin stimulation measured by RFI (normalized to GFP^- cells) on UT, edited (GFP^+) or unedited (GFP^-) male HD $CD4^+$ T cells. Cells were edited with donor templates (from left to right): (i) containing a wild-type cDNA or a codon-usage optimized cDNA (n = 2 UT, 6 WT, 6 CO, 12 GFP^-); (ii) with or without one or two short intervening introns (n = 2 UT, 4 CO+1 intron, 4 CO+2 introns, 4 CO, 12 GFP^-); (iii) with or without CD40LG downstream enhancer sequence (n = 1 UT, 1 CO+enh, 2 CO, 3 GFP^-); and (iv) containing two different polyA signals (n = 2 UT, 6 endogenous CD40LG, 6 SV40 polyA, 12 GFP^-). For statistical analysis, for each case (i or iv), longitudinal comparisons were performed with an LME model, accounting for multiple donors, followed by an appropriate *post hoc* analysis (see Appendix Supplementary Statistical Methods). For the analysis, UT group was not considered due to the small sample size. The reported statistical comparisons refer only to 8-h time-point (**P = 0.0049 and ****P < 0.0001 in all comparisons). Median ± IQR.
K Percentage of NHEJ at 17 days after *CD40LG* editing in male HD T cells, measured by mismatch-sensitive endonuclease assay. Cells were edited with multiple doses of RNP (0.62, 1.25, 1.87 μM) or AAV6 (10^4, 5×10^4) (n = 2 for each group). Median.
L Percentage of GFP^+ cells within $CD4^+$ and $CD8^+$ subpopulations in T cells from (K) (n = 2 for each group). Median ± Range.
M Percentage of GFP^+ cells or HDR in T cells from (K) (n = 2 for each group). Median ± Range.
N Growth curve in culture of T cells from (K) (n = 2 for each group). Median ± Range.
O Schematics of 3′UTR HDR integrations and gDNA analysis of 3′UTR HDR from edited (bulk), non-edited (GFP^-), and UT $CD4^+$ T cells. Primer location is depicted with red arrows.
P Representative plot showing CD40L and GFP expression in bulk edited $CD4^+$ T cells derived from patient carrying exon 1 mutation at 8 h after PMA/Ionomycin stimulation.
Q Left: FACS plots showing percentage of class-switched B cells when co-cultured with HD or Pt T cells from Fig 1K and L. Right: Average percentage of class-switched B cells (IgG^+) resulting from B–T cell co-culture (n = 1 for each group). B cells cultured alone (–) or in presence of sCD40L (+) were used as negative and positive controls, respectively.

© 2021 The Authors

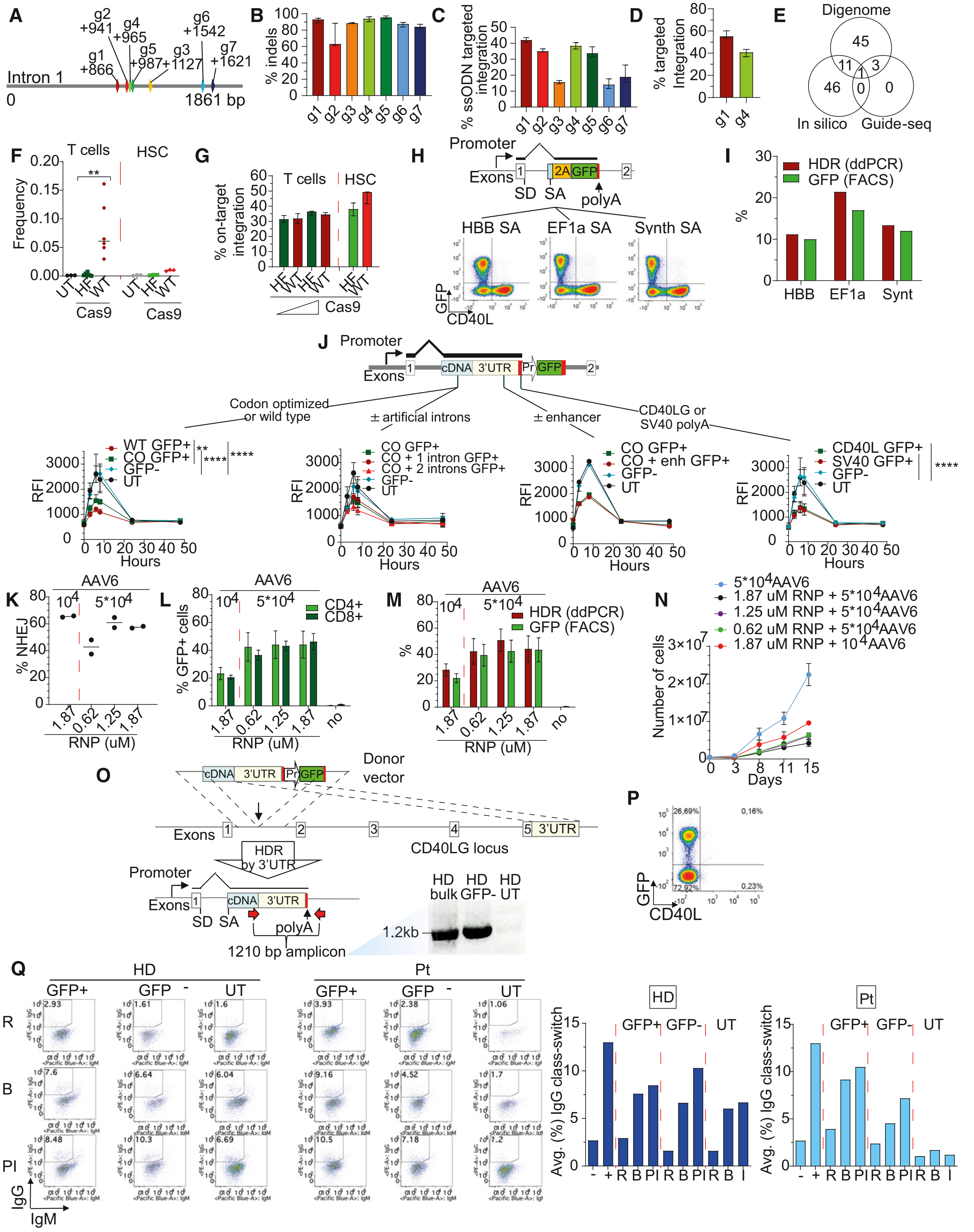
A
Intron 1
0
1861 bp
g1 +866
g2 +941
g4 +965
g5 +987
g3 +1127
g6 +1542
g7 +1621
B
% indels
C
% ssODN targeted integration
D
% targeted Integration
E
Digenome
In silico
Guide-seq
45
11
3
46
1
0
0
F
T cells
HSC
Frequency
UT HF WT
Cas9
G
% on-target integration
T cells
HSC
Cas9
H
Promoter
Exons
SD SA
polyA
2A GFP
HBB SA
EF1a SA
Synth SA
GFP
CD40L
I
HDR (ddPCR)
GFP (FACS)
HBB EF1a Synt
J
Promoter
Exons
cDNA 3'UTR Pr GFP
Codon optimized or wild type
± artificial introns
± enhancer
CD40LG or SV40 polyA
WT GFP+
CO GFP+
GFP-
UT
CO + 1 intron GFP+
CO + 2 introns GFP+
CO + enh GFP+
CD40L GFP+
SV40 GFP+
RFI
Hours
K
AAV6
% NHEJ
RNP (uM)
L
% GFP+ cells
CD4+
CD8+
M
HDR (ddPCR)
GFP (FACS)
N
5*10^4 AAV6
1.87 uM RNP + 5*10^4 AAV6
1.25 uM RNP + 5*10^4 AAV6
0.62 uM RNP + 5*10^4 AAV6
1.87 uM RNP + 10^4 AAV6
Number of cells
Days
O
Donor vector
CD40LG locus
HDR by 3'UTR
1210 bp amplicon
HD bulk
HD GFP-
HD UT
1.2kb
P
26,69%
0,16%
72,92%
0,23%
GFP
CD40L
Q
HD
Pt
GFP+
GFP-
UT
R
B
PI
IgG
IgM
Avg. (%) IgG class-switch

Figure EV1.

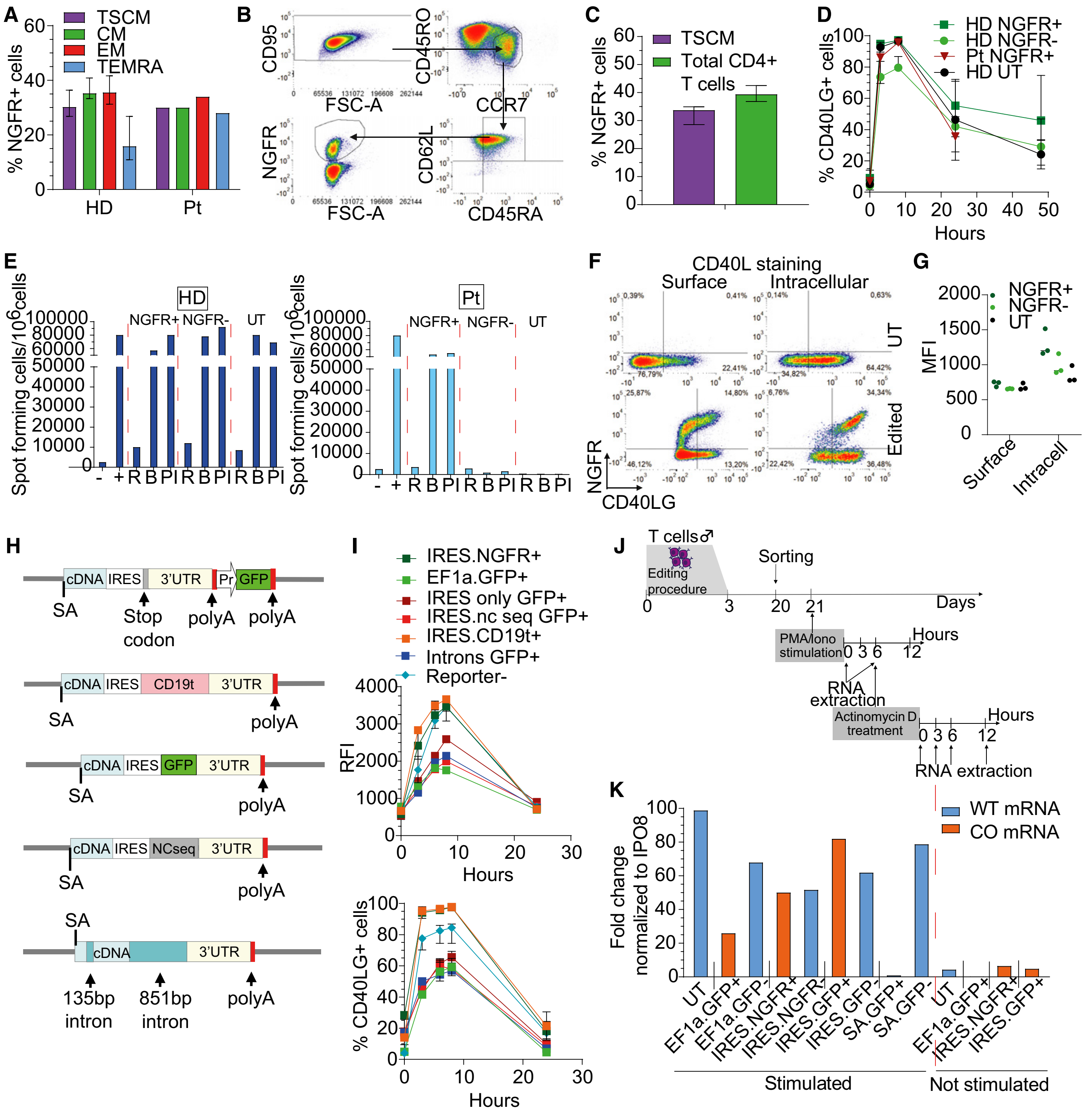

Figure EV2.

 © 2021 The Authors

Figure EV2. Investigation of mechanisms underlying functional and complete rescue of CD40L expression.

A Percentage of NGFR$^+$ cells within T-cell subpopulations at 17 days after *CD40LG* editing of male HD (n = 10) or patient (Pt; n = 1) derived CD4$^+$ T cells, measured by FACS analysis. Median ± IQR.

B Representative plots showing more detailed characterization of edited TSCM cells, defined as CD95$^+$CCR7$^+$CD45RO$^+$CD62L$^+$CD45RA$^+$.

C Percentage of NGFR$^+$ cells within TSCM cell subpopulation (CD95$^+$CCR7$^+$CD45RO$^+$CD62L$^+$CD45RA$^+$) or within total live cells, 17 days after *CD40LG* editing of healthy male donor (HD; n = 3) derived CD4$^+$ T cells, measured by FACS analysis. Median ± Range.

D Time course of CD40L surface expression after PMA/Ionomycin stimulation measured by percentage on UT (n = 2), edited (NGFR$^+$) or unedited (NGFR$^-$) HD or Pt derived CD4$^+$ T cells (n = 4 HD, 1 Pt) T cells from Fig 2C. Median ± IQR.

E IgG$^+$ secreting B cells, evaluated by ELISPOT assay. B cells were isolated from PB of HD and co-cultured with male HD (left) or Pt (right) sorted NGFR$^+$, NGFR$^-$, and UT T cells, resting (R) or stimulated with beads (B) or PMA/Ionomycin (PI). B cells cultured alone (−) or in presence of sCD40L (+) were used as negative and positive controls, respectively (n = 1 for each group).

F Representative plots showing CD40L expression after surface (left) or intracellular staining (right) in UT or bulk edited CD4$^+$ T cells derived from male HD in absence of PMA/Ionomycin stimulation.

G CD40L expression measured by MFI after surface or intracellular staining in UT or bulk edited CD4$^+$ T cells derived from male HD in absence of PMA/Ionomycin stimulation (n = 3). Median.

H Schematics of donor DNA templates used across experiments shown in Fig 2I and J and in (I, K). Corrective *CD40LG* cDNA was coupled to (i) IRES sequence alone (IRES.only), (ii) IRES followed by a reporter gene other than NGFR (CD19 or GFP; IRES.CD19t or IRES.GFP), IRES followed by a non-coding sequence (IRES.nc seq), or engineered with two long intervening introns (Introns). Donor templates carrying *CD40LG* cDNA followed by GFP reporter cassette (EF1a.GFP) or coupled to NGFR by IRES sequence (IRES.NGFR) were used as controls.

I Time course of CD40L surface expression after PMA/Ionomycin stimulation measured by RFI (normalized to Reporter$^-$ cells; upper panel) and percentage (lower panel) on edited or unedited (Reporter$^-$; n = 11) male HD CD4$^+$ T cells. Cells were edited with constructs depicted in (H) (n = 2 IRES.only, 2 IRES.nc seq, 2 IRES.CD19t, 1 Introns, 2 EF1a.GFP, 2 IRES.NGFR). Median ± IQR.

J Flow chart of experiment reported in Fig 2I and J and in (K).

K Bar plot depicting expression of *CD40LG* wild-type mRNA (WT) or *CD40LG* edited mRNA (codon-usage optimized, CO) measured as fold change (FC) on IPO8 housekeeping gene. UT, sorted edited (+) and sorted unedited (−) CD4$^+$ T cells were analyzed before (not stimulated) and 6 h after PMA/Ionomycin stimulation. Cells were edited with donor templates from (K) and donor template from Fig EV1H (SA.GFP; *CD40LG* knockout) was used as negative control. Representative experiment shown out of 3.

© 2021 The Authors

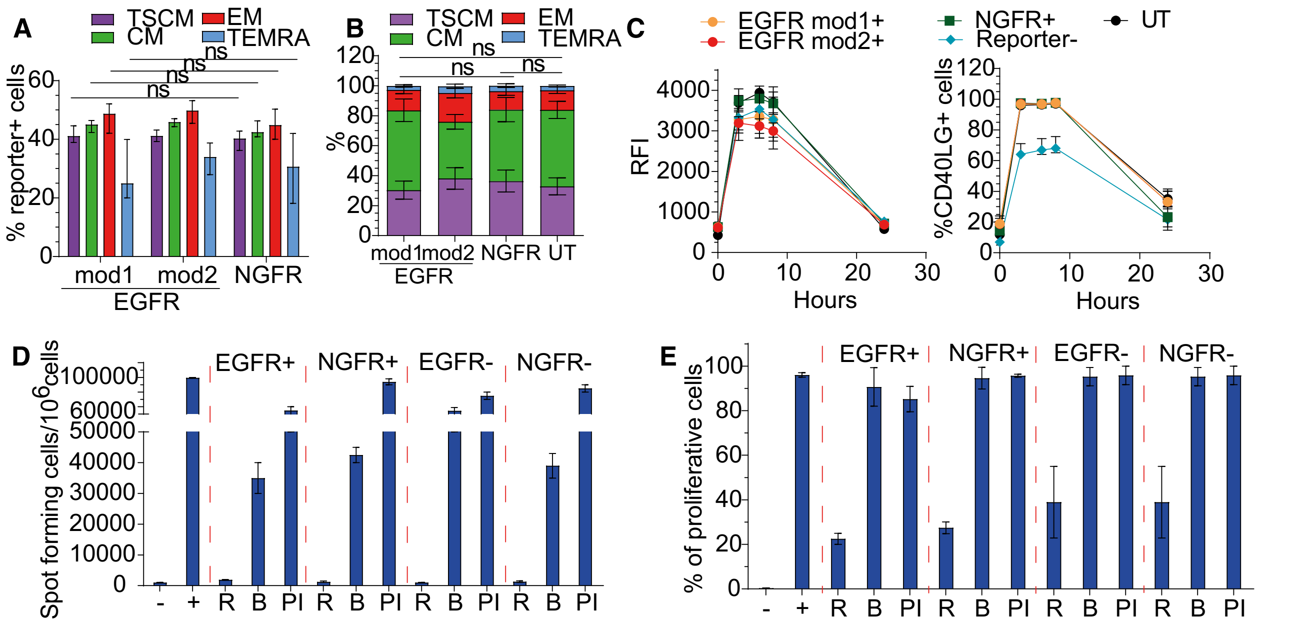

Figure EV3. Characterization of T cells edited exploiting clinically compliant selector hEGFRt.

A Percentage of Reporter$^+$ cells within T-cell subpopulations at 17 days after *CD40LG* editing in male HD-derived CD4$^+$ T cells, measured by FACS analysis. Cells were edited with donor templates carrying NGFR (n = 7), EGFRmod1 (n = 7), or EGFRmod2 (n = 4). Paired Wilcoxon's test (to account for the same donor) with *P*-values adjusted with Bonferroni's correction to account for multiple testing. EGFRmod2 was not included in the analysis because n = 4. Median ± IQR.

B Population composition in male HD-derived UT T cells (n = 14) or bulk edited T cells from (A). Comparisons were performed by estimating an LME model for each subpopulation, accounting for the same donor and different replicates per donor within group, followed by a *post hoc* analysis performed with the R package phia. *P*-values were adjusted with Bonferroni's correction for both multiple testing and multiple comparisons. In the analysis of the subpopulations, TSCM and TEMRA, the percentages were used in natural logarithmic scale to meet the assumption of normality of the residuals of the models. EGFRmod2 was not included in the analysis because n = 4. Mean ± SEM.

C Time course of CD40L surface expression after PMA/Ionomycin stimulation measured by RFI (normalized to Reporter$^-$ cells; left) and percentage (right) on UT, edited, or unedited T cells from (A) (n = 4 for each group, except for Reporter$^-$ n = 12). Median ± IQR.

D IgG$^+$ secreting B cells, evaluated by ELISPOT assay. B cells were isolated from PB of HD and co-cultured with male HD sorted NGFR/EGFR$^+$ and NGFR/EGFR$^-$, resting (R) or stimulated with beads (B) or PMA/Ionomycin (PI). B cells cultured alone (–) or in presence of sCD40L (+) were used as negative and positive controls, respectively (n = 2 for each group). Mean ± SEM.

E Analysis of B-cell proliferative capacity by Cell Trace dilution assay in allogeneic sorted B cells isolated from PB of HD and co-cultured with male HD T cells from (D). B cells cultured alone (–) or in presence of sCD40L (+) were used as negative and positive controls, respectively (n = 2 for each group). Mean ± SEM.

 © 2021 The Authors

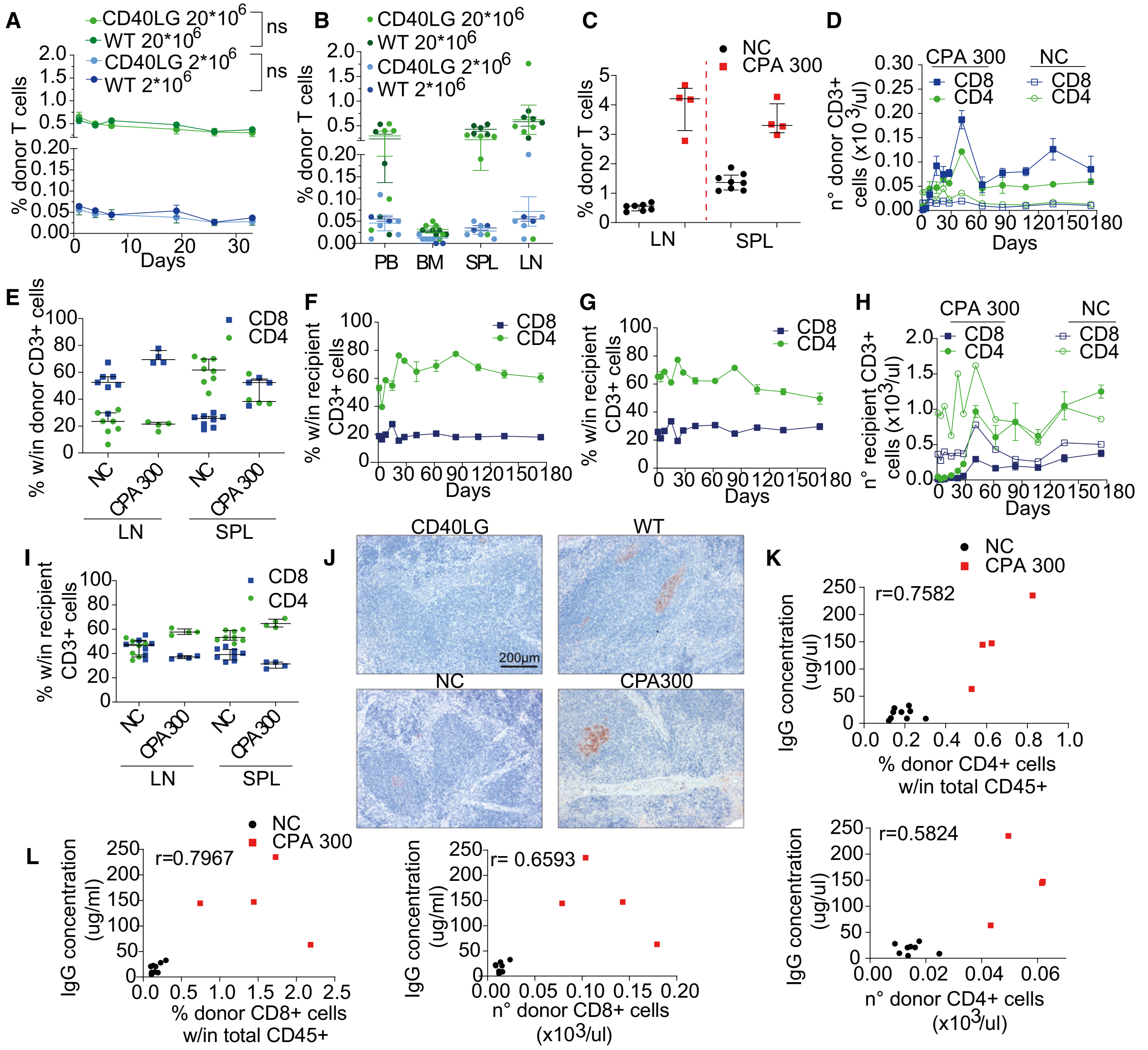
A
CD40LG 20*10^6
WT 20*10^6
CD40LG 2*10^6
WT 2*10^6
ns
ns
% donor T cells
Days
B
PB
BM
SPL
LN
C
NC
CPA 300
LN
SPL
D
n° donor CD3+ cells (x10^3/ul)
CD8
CD4
Days
E
% w/in donor CD3+ cells
F
% w/in recipient CD3+ cells
G
H
n° recipient CD3+ cells (x10^3/ul)
I
J
CD40LG
WT
200μm
CPA300
K
r=0.7582
IgG concentration (ug/ul)
% donor CD4+ cells w/in total CD45+
L
r=0.7967
IgG concentration (ug/ml)
% donor CD8+ cells w/in total CD45+
r= 0.6593
n° donor CD8+ cells (x10^3/ul)
r=0.5824
n° donor CD4+ cells (x10^3/ul)

Figure EV4.

Figure EV4. Analysis of mice transplanted with syngeneic naive T cells.

A Percentage of engrafted donor $CD45.1^+$ cells after adoptive transfer of 2×10^6 or 20×10^6 spleen derived WT $CD3^+$ T cells in PB of CD45.2 *Cd40lg*$^{-/-}$ ($n = 5$ CD40LG 2×10^6, 5 CD40LG 20×10^6) or CD45.2 WT mice ($n = 5$ WT 2×10^6, 5 WT 20×10^6). Longitudinal comparisons between CD40LG and WT were performed with an LME model followed by an appropriate *post hoc* analysis, separately for each dose (see Appendix Supplementary Statistical Methods). For 2×10^6 data, the time-points 19, 26, and 33 days were not analyzed since $n = 3$ for WT. Mean ± SEM.

B Percentage of engrafted donor $CD45.1^+$ cells in PB, bone marrow, spleen, and lymph nodes of mice from (A). Mean ± SEM.

C Percentage of engrafted donor $CD45.1.2^+$ cells in spleen and lymph nodes of mice from Fig 5B ($n = 4$ CPA 300, 8 NC). Median ± IQR.

D Total counts of donor $CD4^+$ and $CD8^+$ cells gated within $CD3^+$ $CD45.1.2^+$ cells in PB of CPA treated or NC *Cd40lg*$^{-/-}$ mice from Fig 5B ($n = 4$ CPA 300, 9 NC). Mean ± SEM.

E Percentage of donor $CD4^+$ and $CD8^+$ cells gated within $CD3^+$ $CD45.1.2^+$ cells in spleen and lymph nodes of mice from Fig 5B ($n = 4$ CPA 300, 9 NC). Median ± IQR.

F, G Percentage of recipient $CD4^+$ and $CD8^+$ cells gated within $CD3^+$ $CD45.2^+$ cells in PB of CPA treated (F) or NC (G) *Cd40lg*$^{-/-}$ mice from Fig 5B ($n = 4$ CPA 300, 9 NC). Mean ± SEM.

H Total counts of recipient $CD4^+$ and $CD8^+$ cells gated within $CD3^+CD45.2^+$ cells in PB of CPA treated or NC *Cd40lg*$^{-/-}$ mice from Fig 5B ($n = 4$ CPA 300, 9 NC). Mean ± SEM.

I Percentage of recipient $CD4^+$ and $CD8^+$ cells gated within $CD3^+CD45.2^+$ cells in spleen and lymph nodes of mice from Fig 5B ($n = 4$ CPA 300, 8 NC). Median ± IQR.

J Representative peanut agglutinin (PNA) immunohistochemical staining of spleen sections from TNP-KLH-immunized mice. Brown (PNA^+) areas represent GC.

K, L Correlation between TNP-KLH-specific IgG concentration in sera and percentage (upper in (K), left in (L)) or total counts (lower in (K), right in (L)) of engrafted donor $CD4^+$ (K) or $CD8^+$ (L) T cells, gated within total $CD45^+$ cells. Spearman's correlation coefficient is reported.

© 2021 The Authors

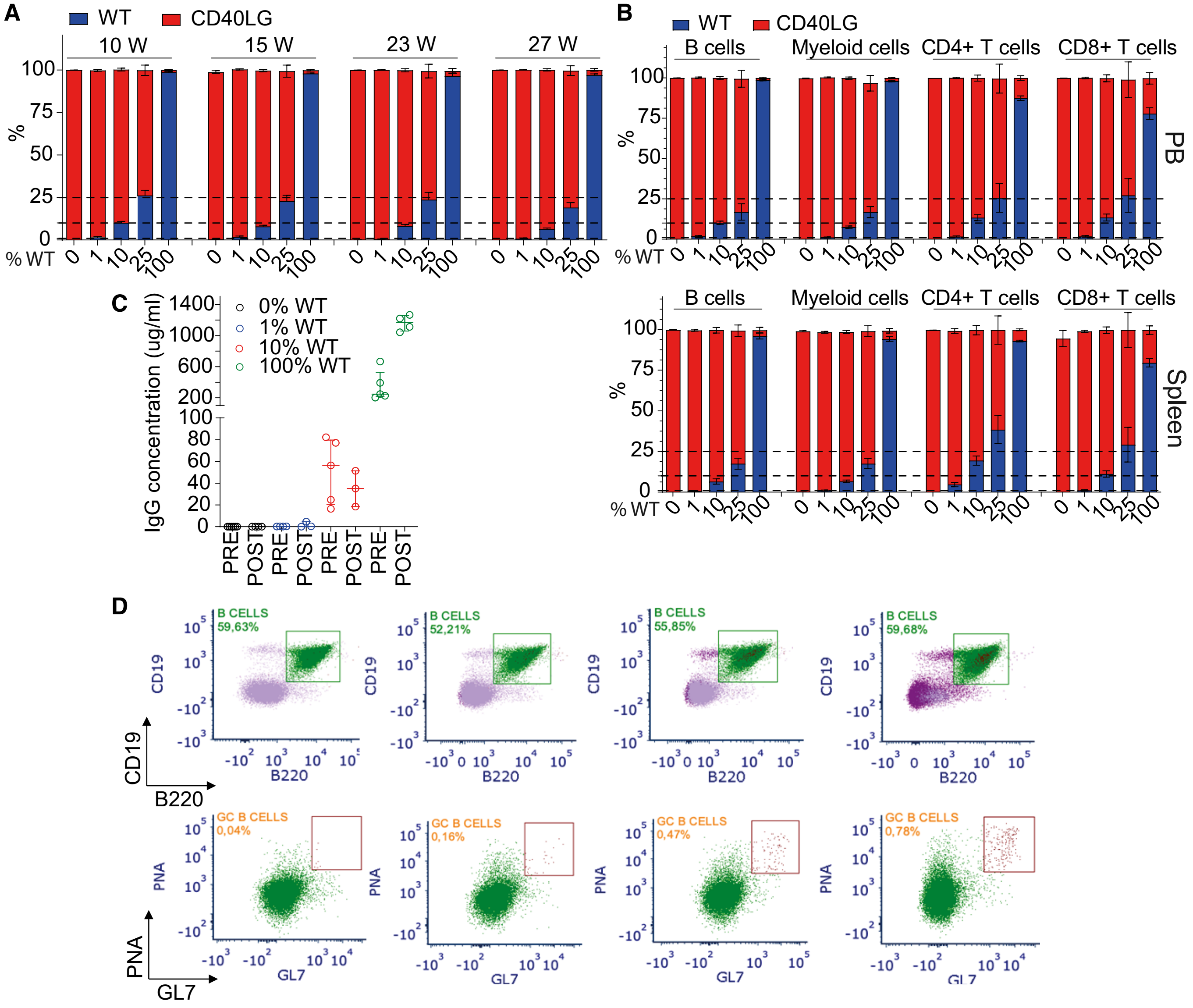

Figure EV5. Partial rescue of the IgG response and GC formation by HSPC therapy.

A Chimerism of WT and *Cd40lg*$^{-/-}$ cells observed within CD11b^{+} myeloid cells at long-term follow-up after transplant in PB of mice from Fig 7A (n = 21 100% WT, 8 25% WT, 22 10% WT, 14 1% WT, 22 0% WT). Three independent experiments. Mean ± SEM.

B Chimerism of WT and *Cd40lg*$^{-/-}$ cells observed within CD19^{+} B cells, CD4^{+} T cells, CD8^{+} T cells and CD11b^{+} myeloid cells at the end of the experiment in PB (upper panel) (n = 20 100% WT, 6 25% WT, 21 10% WT, 14 1% WT, 21 0% WT) or spleen (lower panel) (n = 18 100% WT, 6 25% WT, 19 10% WT, 13 1% WT, 20 0% WT) of mice from Fig 7A. Three independent experiments. Mean ± SEM.

C OVA-specific IgG concentration in sera of mice from Fig 7A collected 7 days before (pre) and after (post) OVA booster immunization (n = 4 100% WT, 5 10% WT, 4 1% WT, 4 0% WT). Median ± IQR.

D Representative plots showing the gating strategy used to characterize B220^{+}PNAhigh GC B-cell percentages from Fig 7A within each experimental group. Splenic mononuclear cells were stained with B220, CD19, PNA, and GL7. PNA^{+}GL7^{+} GC B cells are derived from B220^{+}CD19^{+} gated splenocytes.

© 2021 The Authors
